# eHealth interventions to support colorectal cancer patients’ self-management after discharge from surgery—an integrative literature review

**DOI:** 10.1007/s00520-023-08191-7

**Published:** 2023-12-06

**Authors:** Anne Lunde Marie Husebø, Jon Arne Søreide, Hartwig Kørner, Marianne Storm, Hege Bjøkne Wathne, Alison Richardson, Ingvild Margreta Morken, Kristin Hjorthaug Urstad, Oda Karin Nordfonn, Bjørg Karlsen

**Affiliations:** 1 Department of Public Health, Faculty of Health Sciences, University of Stavanger, 4036 Stavanger, Norway; 2 Research Group of Nursing and Health Sciences, Research Department, Stavanger University Hospital, Stavanger, Norway; 3 Department of Gastrointestinal Surgery, Stavanger University Hospital, Stavanger, Norway; 4 Department of Clinical Medicine, University of Bergen, Bergen, Norway; 5 Faculty of Health Sciences and Social Care, Molde University College, Molde, Norway; 6 NIHR CLAHRC Wessex, School of Health Sciences, University of Southampton, Building 67, Highfield Campus, University Road, Southampton, SO17 1BJ UK; 7 University Hospital Southampton NHS Foundation Trust, Southampton General Hospital, Mailpoint 11, Clinical Academic Facility (Room AA102), South Academic Block, Tremona Road, Southampton, SO16 6YD UK; 8 Department of Quality and Health Technologies, Faculty of Health Sciences, University of Stavanger, 4036 Stavanger, Norway; 9 Faculty of Health Studies, VID Specialized University, Oslo, Norway; 10 Department of Health and Caring Science, Western Norway University of Applied Science, Stord, Norway

**Keywords:** Colorectal cancer, eHealth intervention, Post-hospital discharge follow-up, Post-primary surgery, Self-management support, Quality of life

## Abstract

**Introduction:**

Colorectal cancer (CRC) creates elevated self-management demands and unmet support needs post-discharge. Follow-up care through eHealth post-primary surgery may be an effective means of supporting patients’ needs. This integrative review describes the evidence regarding eHealth interventions post-hospital discharge focusing on delivery mode, user-interface and content, patient intervention adherence, impact on patient-reported outcomes and experiences of eHealth.

**Methods:**

A university librarian performed literature searches in 2021 using four databases. After screening 1149 records, the authors read 30 full-text papers and included and extracted data from 26 papers. Two authors analysed the extracted data using the ‘framework synthesis approach’.

**Results:**

The 26 papers were published between 2012 and 2022. The eHealth interventions were mainly delivered by telephone with the assistance of healthcare professionals, combined with text messages or video conferencing. The user interfaces included websites, applications and physical activity (PA) trackers. The interventions comprised the monitoring of symptoms or health behaviours, patient information, education and counselling. Evidence showed a better psychological state and improved PA. Patients reported high satisfaction with eHealth. However, patient adherence was inadequately reported.

**Conclusions:**

eHealth interventions may positively impact CRC patients’ anxiety and PA regardless of the user interface. Patients prefer technology combined with a human element.

## Background

Colorectal cancer (CRC) is the third most common cancer globally [1]. Cancer stages I–III (i.e. nonmetastatic disease) dominate among CRC cases, with curative surgery being the cornerstone of treatment [2]. Patients with CRC are prone to comorbidities [3]. The impact of surgery, in combination with comorbidities, is found to be highest in the first year following surgery [4]. Most CRC patients are currently managed within enhanced recovery schemes [5], including early discharge to home post-surgery, when physiological functions such as oral intake of nutrients or bowel functions may not be fully restored [6]. About half of anastomotic leakages after bowel resection occur after discharge from hospital, with serious consequences for the patient [7]. Consequently, the period of transition from hospital to home may represent a vulnerable time, prone to issues that can contribute to readmission. Readmission rates for CRC range from 9 to 25% [8] and are deemed markers of quality of care [9].

Following discharge, many CRC patients may struggle with navigating the healthcare system and adopting recommended self-management behaviours. The self-management of CRC includes monitoring health, accessing health information [10] and initiating health behaviour changes, such as exercising more [11]. Moreover, CRC patients may struggle with self-management tasks like finding medical information, monitoring health and interacting with healthcare services, which may result in physical and mental fatigue [10].

eHealth is defined as ‘the use of information and communication technologies (ICT) for health’ [12]. eHealth support deployed post-hospitalisation may promote self-management among people with severe conditions [13]. However, further insight is needed into how a more seamless eHealth service during the transition from inpatient to outpatient care may enable patients to obtain adequate self-management support, feel safe and recover well [14].

There is some evidence that eHealth can support cancer survivors in the self-management of treatment side effects and complications and increase their quality of life (QOL) [15]. Recent reviews of eHealth in the context of CRC populations are sparse. In an overview of reviews on telemedicine (e.g. eHealth) in post-treatment cancer survivorship, none of the 29 included systematic reviews focused on CRC patients only [16]. A systematic review aiming to study eHealth support directed at CRC survivors’ follow-up needs upon discharge from the hospital addressed the interventions’ service content, outcomes and software infrastructure [17]. The findings demonstrated that eHealth was useful for CRC survivors in supporting physiological, psychological and cognitive needs and enabling better symptom management and QOL [17]. Nevertheless, there is a knowledge gap concerning technology acceptance and how patients adhere to eHealth interventions. Adherence is defined as ‘the extent to which a person’s behaviour corresponds with agreed recommendations from a healthcare provider’ [18] (p. 3), but little is known about how eHealth may promote adherence to recommended CRC self-care [19].

This study aimed to (1) explore the user interface, content and delivery mode of CRC eHealth interventions following discharge after surgery, (2) investigate patient adherence to the interventions, (3) establish intervention effects on patient-reported outcome measures (PROMs) and (4) describe patients’ experiences of eHealth follow-up interventions.

## Methods

The study was conducted according to Whittemore and Knafl’s five-step framework for integrative reviews [20] and the Preferred Reporting Items for Systematic Reviews and Meta-Analyses (PRISMA) [21].

### Step 1: literature search

Comprehensive literature searches were performed by a university librarian in October 2021 using the Embase, Medline, CINAHL and Cochrane Library databases, as well as by manually searching reference lists. The search terms, limitations and search results are displayed in Table 1.

**Table 1:** Searches in library electronic databases

Search no.	Combination of search terms	Limitations	Number of records identified
1	Colorectal or colon or rectal or sigmoid or sigmoid colon or colon sigmoid or mesocolon, cancer or carcinoma or neoplasm or tumour or tumour	English language	786 783
2	Terms telehealth or tele-health or teleme* or tele-med or ehealth or e-health or mhealth or m-health or mobile health or teleconsult or tele-consult or telenursing or tele-nursing or telepatholog or tele-patholog or telerehab or tele-rehab or videoconsult or video-consult or digital health, video or webcam or virtual or tele or digital or e-mail or email or remote or electronic or online or mobile, visit or appointment or consult or rehab or counsel or therap or educat	English language	259 328
3	Search no. 1 and Search No. 2	English language	1573

###  Step 2: study selection

Endnote™ Version X9 [22] was used to manage the generated records from Search no. 3 (Table 1). After removing duplicates (*N*=471), a blinded screening of 1373 titles and abstracts was performed using the web application Rayyan [232] and *a priori* inclusion and exclusion criteria (Table 2). Following the blinded screening, a comparison of the decisions showed discrepancies for 37 records (4.5%), resolved through discussions among the authors. Fifty-nine full-text articles were distributed among the authors and assessed for final inclusion, with conflicting opinions being resolved through discussions among the authors. The results of the study selection process are displayed in a PRISMA flow chart [23].

**Table 2:** Inclusion and exclusion criteria

Inclusion criteria	Exclusion criteria
Original and empirical studies using quantitative, qualitative or mixed methods	Reviews, syntheses, meta-analyses, book chapters, study protocols, conference papers, grey literature
Adult patient populations with CRC	Child and adolescent patients
eHealth interventions to support CRC patients following primary surgery	eHealth studies targeting CRC patients in the pre-surgical phase Studies on screening or surveillance of the development of CRC eHealth studies not reporting on CRC patient outcomes Studies reporting on the development phase of eHealth programs
Peer-reviewed studies	
Published in English between 2012 and 2022	

###  Step 3: data extraction

To achieve consistency in data extraction, an extraction tool was constructed, including publication identifiers, study design, study context and participants, eHealth program, program adherence and patient outcomes and experiences. Any inconsistencies among co-authors were resolved via the assessment of a second reviewer.

### Step 4: critical assessment of articles

The authors used the mixed methods appraisal tool (MMAT) [24] in teams of two to establish the risk of bias in the included studies. Here, the MMAT checklists for randomised controlled trials (RCTs) and non-randomised, descriptive, qualitative and mixed methods were used. Each study was assigned an overall quality score, varying from 25% when one criterion was met to 100% when all criteria were met. The MMAT was used as a summarising tool, with methodological quality considered according to the design of each study. The MMAT score was not used for exclusion decisions [24]. Studies were not excluded based on methodological quality. The strength of evidence was summarised as part of the review’s limitations.

###  Step 5: data synthesis

To analyse and synthesise data, the ‘framework synthesis approach’ was used, which includes five analytical stages: familiarisation with the data content, identification of themes, indexing, charting and mapping and interpretation [25]. Data from the extraction table allowed the authors to familiarise themselves with the findings. Coding of the data was performed by one author according to key issues, concepts and themes, namely the outcomes and practices of eHealth follow-up programs, including content, delivery mode and user interface, patient adherence, impact of eHealth interventions and patient experience. The synthesis of the findings was then reviewed by a second author and finally examined by the co-authors.

## Results

**Fig. 1:**
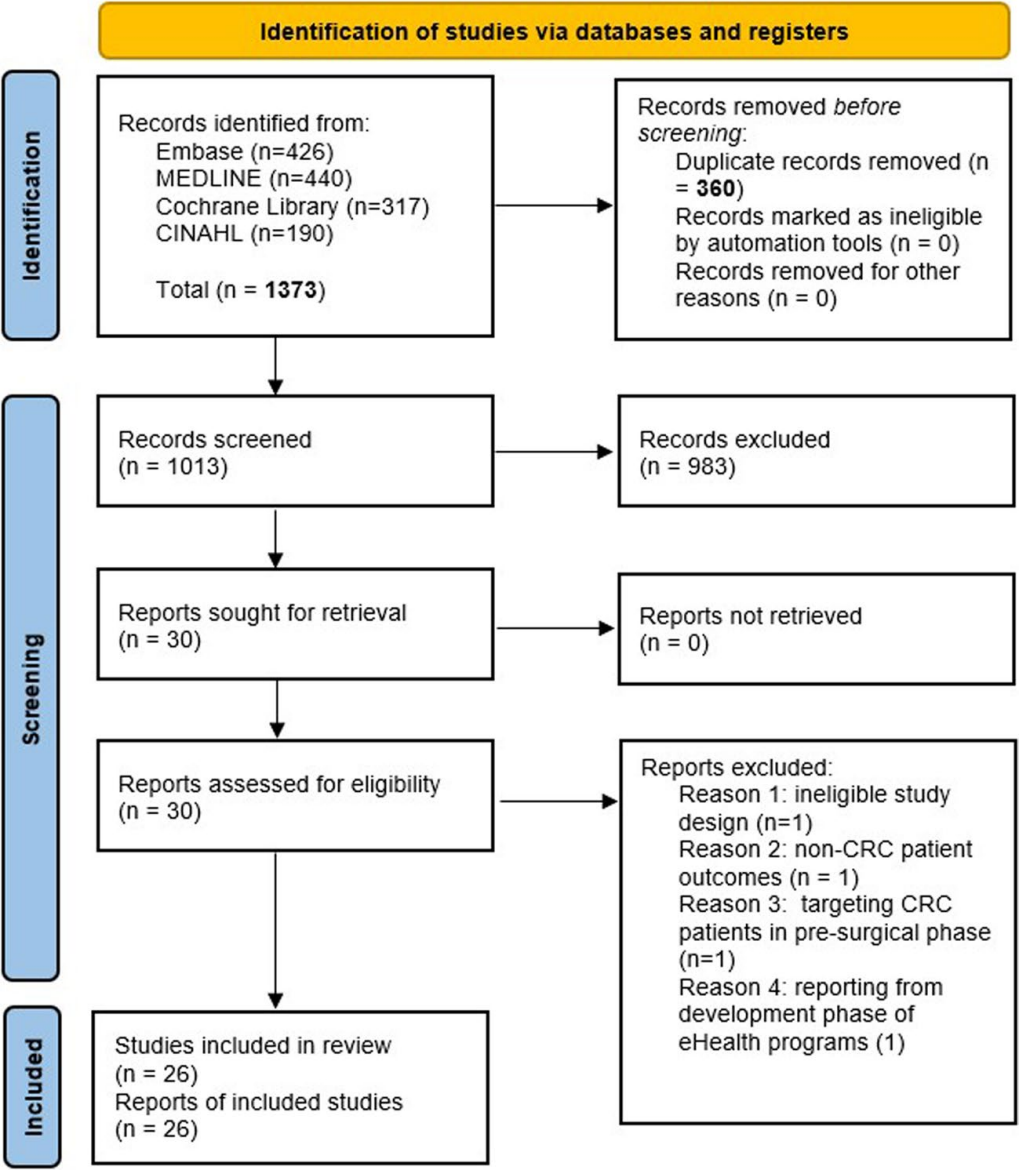
PRISMA flowchart of study selection process

After the full-text assessment of the 30 records, four were excluded based on the eligibility criteria resulting in a total of 26 included papers [26–51] (Fig. 1).

### Risk of bias

Among the 12 RCTs, two achieved full scores (7/7 points) [30, 51], four achieved 6/7 points [35, 42, 46, 49], five scored 5/7 points [38, 40, 43, 47, 48] and one scored 4/7 points [28]. Across the MMAT domains, seven of 12 RCTs did not report the blinding of outcome assessors. Nearly all the non-randomised studies scored 5/6 points, while one achieved 4/6 points [26]. Here, the less reported item referred to administration of the intervention as intended (5/8 studies). Only one of the three descriptive achieved the full score of 7 points [45] as the authors of the other two did not report on sample representativeness, risk of non-response bias and appropriate statistical analysis [33, 44]. The only mixed-methods study scored 5/7 points as it lacked reporting on sample representativeness and an adequate rationale for using a mixed-methods design to address the research question [36]. Both qualitative studies [31, 50] demonstrated good methodological quality, scoring 7/7 points.

### Overview of study characteristics

The studies were published between 2012 and 2022 (Table 3), and most were of European origin. Three studies were performed by the same Swedish research team [31–33], while three Dutch studies involved the same eHealth application (i.e. Oncokompas) [47–49]. Fourteen studies applied RCT or quasi-experimental study methods, seven used observational designs, two were qualitative, two used mixed methods, and one used a case-study design. The study populations ranged from 1 to 756 participants (median number of participants, *n*=118). In one study, presented in two publications, the CRC patient population accounted for 25% of the participants [47, 48]. All studies recruited adult CRC patients (18–81 years of age). Median age, based on 21 of 26 studies that provided information on mean or median age was 65 years of age [26–39, 41–44, 46–49, 51]. In four studies, most of the participants were female [29, 30, 35, 41, 42]. Only two studies addressed the importance of a diverse sample as to provide eHealth services to demographically (e.g. education and income) and geographically (e.g. rural areas) diverse groups [42, 46]. In all the studies, patients were enrolled during the post-operative care trajectory. In eight studies, patients received the eHealth intervention during adjuvant chemotherapy [26, 28–33, 38, 39].

**Table 3:** A summary of findings on included studies’ origin, publication year, study design, study participants, patient outcomes, patient adherence, findings and quality assessment score

Author (year)	Country	Study design	Study participants	Patient outcomes and measurements	Patient adherence to eHealth interventions	Findings	MMAT score/maximum score
Avci et al. 2018) [26]	Turkey	Quasi-experimental study	Patients receiving adjuvant treatments Age (mean) Intervention group (IG): 59.0 ± 11.5 Control group (CG): 61.6 ± 12.3 Male IG: 75% CG: 68%	Chemotherapy symptoms Anxiety Primary outcome (PO) not stated	Adherence not addressed	The IG experienced significantly less frequently chemotherapy side effects compared with the CG (*p* = < .05), and fewer severe side effects of infection, hair loss, and mouth and throat problems (*p* = < .05). Anxiety decreased significantly in the IG compared with controls (*p* = > .001)	4/6
Barsom et al. 2021) [27]	The Netherlands	Observational study	Patients at outpatient clinic follow-upAge (mean) IG: 68 (SD=57-74) CG: 61 (SD=53-69) Male IG: 39% CG: 52%	Patient attitudes towards video consultations (VC) Patients’ reporting of usability Patient satisfaction with interaction with healthcare provider Provider satisfaction with the consultation PO not stated	Adherence not addressed	VC-group expressed highly positive attitude in using VC. Face to face-group (F2F) was less concerned with privacy issues. 96% in the VC-group and 38% in the F2F-group would like to use VC in the future. VC-group reported VC easy to use and convenient and would not change to F2F consultations. Usability of VC was rated as excellent by 67%, and good by 33%.	5/6
Beaver et al. 2021) [28]	England	Randomised controlled trial (RCT)	Patients at completion of treatmentAge (mean) IG: 72.4 ± 8.2 CG: 73.6 ± 7.6 Male IG: 64% CG: 52%	Anxiety (PO) General health (PO) Satisfaction with information (PO) Clinical investigations ordered Time to detection of recurrent disease Costs to patients	Adherence not addressed	The telephone intervention decreased anxiety levels. Considerable fewer information needs were raised by the controls at follow-up, compared with intervention participants (16 vs. 30). Intervention participants were more satisfied with the latest appointment. There was no difference between groups concerning contact with healthcare services during the study. The telephone appointments lasted significantly longer (*p*=.001), (29 vs.14 minutes).	4/7
Cheong et al. 2018) [29]	South Korea	Observational study	Patients receiving adjuvant treatments Age (mean): 58.27 ± 11.74 Male: 59%	Physical activity (PA) Nutritional status Quality of life (QOL) Physical performance Distress PO not stated	Adherence addressed as the number/percentage of patients who completed the program	Lower extremity strength (*p*<0.001) and cardiorespiratory endurance (*p*<0.001) improved. Fatigue (*p*=0.007) and nausea/vomiting (*p*=0.04) were relieved.	5/6
Dong et al. (2019) [30]	China	RCT	Patients receiving adjuvant treatments Age (mean) Total sample: 59.09 ± 8.07 Male IG (telephone-based reminiscence (TBR) group): 53% Female IG (telephone-support (TS) groups): 53% CG: 51%	Anxiety Subjective well-being Social support PO not stated	Adherence not addressed	SDS and HAMD scores decreased significantly in the TBR group, but not in the CG and TS groups (*p*<0.05); however, no significant post-intervention scores between TS and TBR groups were found. Neither TS nor TBR improved subjective well-being of social support.	7/7
Drott et al. (2016) [31]	Sweden	Qualitative study	Patients post-adjuvant treatments Age (median, range): 65 (44-68) 4 men and 7 women	Patients’ experiences of using the mobile phone-based system for reporting neurotoxic side effects	Not applicable	The patients’ experiences were identified as (1) being involved, (2) pacing oneself and (3) managing questions. The mobile phone-based system reinforced patients’ feelings of involvement in own care. They were comfortable with the technology and the system was not time consuming.	7/7
Drott et al. (2019) [32]	Sweden	Prospective longitudinal study	Patients receiving adjuvant treatments Age (mean): 61.0 (SD=10) Male: 61%	Severity, frequency and impact of oxaliplatin-associated neurotoxicityPO not stated	Adherence addressed as the response rate to symptom surveys	All patients reported side effects, and severe impact from side effects on daily living activities, with tingling in upper extremities as the most reported. Neurotoxicity symptoms changed significantly from baseline to follow-up in both upper (*p*=.004–.031) and lower extremities (.008–.016).	5/6
Drott et al. (2020) [33]	Sweden	Prospective descriptive cohort study	Patients receiving adjuvant treatments Age (mean): 62.0 (SD=8) Male: 63%	Sense of coherence (SOC) Health-related quality of life (HRQoL) Severity, frequency, and impact of oxaliplatin-associated neurotoxicity PO not stated	Adherence addressed as the response rate to symptom surveys	Neurotoxicity as described in Drott (2018). SOC and overall HRQoL was stable but decrease of social well-being after 1-year follow-up. *p* values not reported.	5/6
Döking et al. (2021) [34]	AustraliaThe Netherlands	Case study	Patient at completion of treatment Age: 74 Male	Psychological distress (PO) Anxiety Fatigue Fear of cancer recurrence Cancer-specific distress Self-efficacy QOL Therapeutic alliance Intervention evaluation	Adherence not addressed	The treatment protocol appeared feasible. Psychological distress showed improved postintervention, while anxiety and cancer-specific distress remained improved during follow-ups. Therapeutic alliance and patient satisfaction were high. Combining face-to-face and online intervention may reduce distress of cancer survivors.	Not screened
Golsteijn et al. (2018) [35]	The Netherlands	RCT	Patients 6 weeks–1-year post-surgery Age (mean) IG: 66.55 (SD=7.07) CG: 66.38 (SD=8.21) Male IG: 85% CG: 89%	Physical activity (PA) behaviour (PO) Fatigue HRQoL Distress	Adherence addressed as minutes of moderate-to-vigorous PA and number of days with 30 minutes or more of PA	Both moderate-to-vigorous PA and days ≥30-min PA increased significantly in the intervention group (*p* =.04 and *p* < .001, respectively). Among secondary outcomes, fatigue and physical functioning improved significantly (*p* = .02 and *p* = .003, respectively). No significant improvement of HRQoL were found. Effects were stronger in CRC patients as compared to prostate cancer patients.	6/7
Grimmett et al. (2015) [36]	United Kingdom	Feasibility study	CRC patient at completion of treatment Age (median, range): 65 (44–79) Female: 62%	PA Diet consumption QOL Fatigue Physical function PO not stated	Acceptability and adherence to counselling sessions Eighteen patients completed all scheduled phone consultations, while five missed one consultation	Significant improvements in objectively measured activity +70 min/pr week (*p*=.004/7) and step counts pr day (*p*=.001). Gains in diet: +3, fruit and vegetable portions a day, (*p*<.001), red meat a week (*p* =.013), and portions of processed meat a week (*p*= .002). Change in serum vitamin levels were was not significant. Significant improvement in quality of life (*p*<.001). *Patient experiences:* patient evaluated phone conversations as mode for iv delivery positive. Several remarked that it was convenient not to have travel. Face to face contact was valued at baseline. Timing of intervention in relation to completion of treatment was considered appropriate. The intervention was considered a helpful and useful exercise.	5/6
Kim et al. (2018) [37]	Korea	Quasi-experimental study	Patients at completion of primary surgery Age (mean) Total sample: 61 (SD=10) Male IG: 64% CG: 58%	QOL (PO) Affective status Depression Self-efficacy Individual resilience	Adherence not addressed	Significant improvement in the IG compared to the CG. Quality of life (*p*=0.0017) Physical status (*p*=0.016) Affective status (*p*=0.0051) Anxiety (*p*=0.0007) Depression (*p*=0.0003) Self-efficacy (*p*=0.0075)	5/6
Li et al. (2019) [38]	China	RCT	Patients receiving adjuvant treatments Age (mean) IG: 60.06 ±11.00 CG: 58.47 ±12.52 Male IG: 66% CG: 61%	Anxiety Depression QOL PO not stated	Adherence not addressed	IG slightly decreased the anxiety grade at M6 compared to the CG (*p*=0.070). The IG had a sig. improvement in depression score M6 versus M0 (*p*<0.001), and the depression grad was reduced in the IG compared to controls (*p*=0.037). QoL, global health status, at M6 versus M0 was increased (*p*=0.0035) and QoL symptom score at M6 versus M0 was decreased (*P*=0.002) in IG versus CG. No difference in QoL, function score, between the groups. Patients in IG had a slight decrease in anxiety and contributed to a significant. reduction in depression and improvement in QoL in CRC patients receiving adjuvant chemotherapy.	5/7
Lin et al. (2014) [39]	Taiwan	Retrospective quantitative study	Patients undergoing treatment Mean/median age not reported Patients’ gender not reported	Patient satisfaction (PO)	Not applicable	43% of the callers were the patients themselves and 37% were the primary caregivers. Some patients called more than once regarding the same condition. Issues: need for emergency treatment (29%), nutrition (21%), chemo side effects (19%), pain (15%). Average calls made by each subject: 0.87 times. Female callers: 66.6% and 43.4% of the calls came on daytime. Average satisfaction level of each question: 90%. Overall satisfaction level: 93%	4/7
Lynch et al. (2014) [40]	Australia	RCT	Patients undergoing treatments (76%) Age: 74% > 60 years Male: 54%	Sedentary behaviour (PO)	Adherence addressed as the percentage of telephone sessions completed	The health coaching intervention showed modest effects on sedentary behaviour. A significant effect on total sedentary time (hours/day) at 12 months was found in CRC survivors aged >60 years, male survivors and in the non-obese.	5/7
Mancini et al. (2021) [41]	Italy	Prospective observational study	Patients at completion of primary surgery Age (median, range): 68 (48–84) Male: 50%	Intervention feasibility and safety (PO) Patient satisfaction	Adherence not addressed	Compliance of patients was > 80%. Overall grade of satisfaction was very high with 4.2 as median (range 0–5). Only two patients were readmitted for surgical consult.	7/7
Mayer et al. (2018) [42]	USA	RCT	Patients at completion of treatment Age (mean) IG: 57.84 (SD=14.5) CG: 59.34 (SD=13.7) Female IG: 51% CG: 52%	PA (PO) Distress QOL	Adherence addressed by defining ‘active users’ of the smartphone application (i.e. creating content or entering or revising data)	No significant differences in PA between the IG and the CG were detected at any timepoint. Both groups went from inactive to moderately active at 6 and 9 months. QoL and distress did not show any significant change over time or between the groups. Both groups reported more physical problems followed by emotional problems.	6/7
Pinto et al. (2013) [43]	USA	RCT	Patients at completion of treatment Age (mean) IG: 59.5 (SD=11.2) CG: 55.6 (8.2) Female 57%	PA Treatment symptoms PO not stated	Adherence not addressed	IG reported significant increases in PA minutes and motivational readiness for PA at 3 months, caloric expenditure, and fitness at 3, 6 and 12 months versus the CG. No significant group differences were found for fatigue, self-reported physical functioning, and quality of life at 3, 6 and 12 months.	5/7
Qaderi et al. (2021) [44]	The Netherlands	Descriptive longitudinal study	Patients at completion of treatment Age (median): 68 (range 63–74) Male 58%	QoL Fear of recurrence Patient satisfaction PO not stated	Adherence addressed as active participation rates	Eighty-three percent of participants reported good, very good or excellent health status. Patient satisfaction at 6 and 12 months scored 7.8 and 7.5 out of 10. After 1 year of follow-up, patients reported advantages of less hospital visits, saved cost and time, increased efficiency and convenience, better access to care and enhanced communication. Disadvantages were loss of human contact and interactive care, and increased threshold to seek help.	5/6
Soh et al. (2018) [45]	Korea	Prospective descriptive study	CRC patients receiving adjuvant treatments Age group In their fifties: 36% Male 63%	Patient satisfaction (PO) QOL	Adherence not addressed	Overall satisfaction rate among subjects was favourable and ranged from 3.93 (SD 0.88) to 4.01 (SD 0.87) on the 5-point Likert scale. ‘Warming-up exercise’ was the most frequently education view. The online survey completion rate was over 40%, and 80% completed the offline survey	7/7
Van Blarigan et al. (2019) [46]	USA	RCT	Patients at completion of treatment Age (mean) IG: 56 ± 12 CG: 54 ± 11 Male 41%	Feasibility and acceptability PA PO not stated	Adherence addressed as Fitbit wear time, response rates to interactive text messages and proportion of participants who completed the 12-week follow-up accelerometer assessment	Among the 16 intervention participants who completed the feedback survey, the majority (88%) reported that the intervention motivated them to exercise and that they were satisfied with their experience. No statistically significant difference in change in moderate-to-vigorous physical activity was found from baseline to 12 weeks between the IG and CG.	6/7
Van der Hout et al. (2020) [47]	The Netherlands	RCT	Patients at completion of treatment Age not reported Gender not reported	Self-management HRQoL PO not stated	Adherence not addressed	Oncokompas did not improve the amount of knowledge, skills and confidence for self-management in cancer survivors. For CRC patients, the course of the symptom weight was significantly different between the intervention and control group (*p* = 0.028).	5/7
Van der Hout et al. (2021) [48]	The Netherlands	RCT	Patients at completion of treatment Age not reported Gender not reported	Patient activation (PO) HRQoL Self-efficacy	Adherence not addressed	Self-efficacy, personal control and health literacy moderated the intervention Oncokompas’ effects on HRQoL (*p*=.034, *p*=.015 and *p*=. o35, respectively)	5/7
Vos et al. (2021) [49]	The Netherlands	RCT	Patients receiving surgical treatment Age (median, range) GP-led group IG: 67 (63-72) CG: 69 (63-75) Surgeon-led group IG: 68 (63-74) CG: 69 (63-75) Male GP-led group IG: 75% CG: 64% Surgeon-led group IG: 63% CG: 67%	QOL (PO) Care coordination Cancer recurrence Self-management Patent satisfaction	Adherence addressed as application use Thirty-six per cent of participants in the Oncokompas group used the app at least once	QoL weas high in all trial groups. At 12 months, there was not clinically meaningful difference in change from baseline in QoL between GP-Led care groups and the surgeon-led care groups or between the Oncokompas and no Oncokompas groups (*p* > .05).	6/7
Williamson et al. (2015) [50]	United Kingdom	Qualitative study	Patients at completion of treatment Age >60 years: 71.4% Male 57%	Patients’ experience with the delivery of the intervention and preference for future technology use	Not applicable	TFU was described as a positive experience and there was a preference for continuing TFU	7/7
Young et al. (2013) [51]	Australia	RCT	Patients receiving surgical treatment Age (Mean, SD) IG: 68.6 (12.2) CG: 67.0 (12.1) Male IG: 57% CG: 54%	Distress Experience of cancer care and supportive care needs Fatigue Patient satisfaction Readmissions PO not stated	Adherence addressed as intervention fidelity based on the proportion of completed calls reported for each time point Call length reported for each time point	There were no significant differences between groups in unmet supportive care needs, emergency department visits or unplanned hospital readmission at 1 month (*p* = > .05). There were no significant differences in experience of care coordination, distress or QoL between groups at any follow-up time point (*p* = > .05). *Patient experiences* Quantitative responses (*n*=350) about the CONNECT nurse and iv were generally positive.	7/7

## Results of data analysis

### eHealth interventions’ delivery mode, user interface and content

The modes of eHealth intervention delivery included telephone (*n*=14) [26, 28, 30, 34, 36–41, 43, 44, 50, 51], websites (*n*=6) [26, 35, 37, 47–49], smartphone applications (*n*=9) [29, 31–33, 37, 41, 42, 45, 46], short message service (SMS; *n*=3) [37, 44, 46] or video consultations (*n*=1) [27] (see Table 4). Several studies combined different modes of delivery. One study supplemented remote follow-up with three home visits during chemotherapy [26]. Of the 26 included studies, the majority involved a delivery mode of direct and analogue contact with a health professional, that is, a nurse [26, 28, 30–33, 37–40, 50, 51], a surgeon/physician/general practitioner [41, 49], a therapist [34] or unspecified research staff [27, 29, 36, 43, 44, 47, 48]. In three studies, the intervention deliverance was purely digital [35, 42, 45, 46].

Patient education and information were included as intervention content in 13 of the eHealth programs, six of which provided education and information on PA behaviour change [29, 35, 36, 42, 43, 46]. In four studies, CRC patients received a digital educational program aiming to strengthen patients’ self-management skills [34, 47–49], while Soh et al. included health education in their mobile care system to support CRC patients’ QOL [45]. Avci et al. provided patients with education and counselling to lower anxiety levels and chemotherapy-based symptoms [26], while Young et al. delivered educational material to meet the emotional needs of CRC patients following surgery [51].

All the intervention studies comprised an element of monitoring of health condition and symptoms. Eight studies monitored the patient’s health condition and treatment side effects by using checklists that the patients responded to electronically [26, 29–31, 35, 39, 41, 44–49]. A pedometer or accelerometer was used to monitor daily PA (e.g. number of steps, walking distance, intensity) in six studies [29, 35, 36, 42, 43, 46], while Cheong et al. applied an activity tracker, like a Fitbit, to monitor the patient’s PA and heart rate [29]. One study used home telemonitoring to follow up with post-operative patients by monitoring vital signs (using an oximeter, thermometer, sphygmomanometer and echocardiogram) and changes in the surgical wound [49]. In one study, CRC patients’ health condition was monitored through a video consultation clinic by the surgeon in charge [27].

Eleven studies used a telephone to provide intervention content comprising counselling, therapy or psychosocial support. Four of those studies offered counselling on managing treatment symptoms and late effects [26, 39], healthy eating [36] or enhancing self-management [37]. Three studies provided supportive calls delivered by nurses focusing on psychosocial support to meet the CRC patients’ emotional and informational needs [27, 38, 50]. Two interventions comprised telephone-delivered reminiscence therapy [30] and cognitive behavioural therapy [34] to reduce mental health symptoms of stress, depression and anxiety. In two studies, health coaching to support PA and dietary issues was part of the telephone-based interventions [40, 43]. In Lynch et al., the eleven 30-min sessions were delivered by nurses, physiologists or health coaches [40], while in Pinto et al., the patients received 12 sessions from research staff based on behavioural cognitive theories to promote exercise self-efficacy [43] (Table 4).

**Table 4:** eHealth interventions’ user interface, delivery mode, content, duration and patient engagement

Author (year)	User interface	Delivery mode	Content	Duration and patient engagement
Avci et al. (2018)	Website Telephone	Tele-counselling Face-to-face home visits Interactive question form	Education, counselling and support	6 months Patient engagement not reported
Barsom et al. (2021)	Video	Tele-counselling	Monitoring Post-surgery counselling	n.a.
Beaver et al. (2021)	Telephone	Delivered by a nurse specialist	Monitoring, support and information	36 months Patient engagement: 8–12 months (mean=12 months)
Cheong et al. (2018)	Smartphone application Wearable device	Real-time communication Delivered by a study coordinator	Information Monitoring	12 weeks Patient engagement: 75 patients participated in 12 weeks
Dong et al. (2019)	Telephone	Delivered by a nurse	Monitoring Support	6 weeks Patient engagement not reported
Drott et al. (2016)	Smartphone application	Delivered by a nurse	Monitoring	12 months Patient engagement: n.a.
Drott et al. (2018)	Smartphone application	Delivered by a nurse	Monitoring	12 months Patient engagement: 70–76% response rate
Drott et al. (2020)	Smartphone application	Delivered by a nurse	Monitoring	12 months Patient engagement not reported
Döking et al. (2021)	Telephone	Delivered by a cognitive behavioural therapist	Monitoring Support Information	4 months Patient engagement not reported
Golsteijn et al. (2018)	Website Accelerometer	n.a.	Monitoring Information	6 months Patient engagement not reported
Grimmett et al. (2015)	Telephone Pedometer Accelerometer	Delivered by researcher	Information Goal setting	3 months Patient engagement: 96% completed the intervention
Kim et al. (2018)	Website on smartphone Text messages Telephone	Delivered by nurses	Education Information	Not reported Patient engagement not reported
Li et al. (2019)	Telephone Workshops	Delivered by nurses	Education Counselling Support Monitoring	6 months Patient engagement not reported
Lin et al. (2014)	Telephone	Delivered by cancer experts	Counselling Support Monitoring	n.a. Patient engagement: 0.87 calls per patient
Lynch et al. (2014)	Telephone Pedometer	Delivered by nurses, psychologists, or health-promotion practitioners	Counselling Support Monitoring	6 months Patient engagement not reported
Mancini et al. (2021)	Smartphone Monitoring devices Telephone Face-to-face sessions	Delivered by surgeons	Monitoring	12 months Patient engagement: 80% completed the intervention
Mayer et al. (2018)	Smartphone application (SurvivorCHESS) Pedometer	n.a.	Information Support Monitoring	6 months Patient engagement: 98% identified as users of the intervention
Pinto et al. (2013)	Telephone Pedometer	Delivered by intervention staff	Support Counselling	3 months Patient engagement: 11.4 calls of 12 completed
Qaderi et al. (2021)	Telephone Personal medical charts Text messages Face-to face sessions	Not reported	Support Information Counselling	34 months Patient engagement not reported
Soh et al. (2018)	Smartphone application Chat	n.a.	Support Education	3 months Patient engagement: 86.7% completed the intervention
Van Blarigan et al. (2019)	Smartphone Text messages Fitbit Flex device	n.a.	Counselling Information	3 months Patient engagement: 74–95% participants in intervention groups completed the intervention
Van der Hout et al. (2020)	Website (I CARE) Application (Oncokompas)	Delivered by helpdesk and healthcare providers	Support Monitoring Information	6 months Patient engagement not reported
Van der Hout et al. (2019)	Website (I CARE) Application (Oncokompas)	Delivered by helpdesk and healthcare providers	Support Monitoring Information	6 months Patient engagement: 52% engaged as intended with the intervention
Vos et al. (2021)	Website (I CARE) Application (Oncokompas)	Delivered by surgeons and general practitioners	Information Support	12 months Patient engagement: 36% engaged with the app once, and 17% > 1 time
Williamson et al. (2015)	Telephone	Delivered by a specialist colorectal cancer nurse	Information	Not reported Patient engagement: n.a.
Young et al. (2013)	Telephone	Delivered by a nurse	Information Support	6 months Patient engagement not reported

### Patient adherence to eHealth follow-up interventions post-hospital discharge

Twelve of the 26 studies did not report on intervention engagement [26–28, 30, 34, 37, 38, 41, 43, 45, 47, 48] (see Table 3). In three studies, reporting on adherence was not applicable due to the study design [31, 39, 50]. In the studies that provided this information, adherence was reported as the participants’ response rates to symptom checklists during and after chemotherapy [30, 32] or home monitoring of vital signs [41], fidelity to the recommended PA sessions [35, 46], response to telephone counselling sessions [26, 36, 39, 40], active use of the eHealth application (e.g. creating content, answering text messages) [37, 42, 46, 49] or completion of the eHealth program [39]. In studies reporting the adherence percentage, adherence was established as 62–100% [36, 39, 41, 47, 48].

### The effects of eHealth interventions on PROMs

A summary of findings on effects from eHealth interventions can be found in Table 3. Sixteen studies included QOL patient outcomes (i.e. health-related quality of life (HRQoL), QOL, subjective well-being, self-efficacy, and sense of coherence) [28–30, 33–38, 42, 44, 45, 47–49]. Only, the study by Kim et al. [35] found strong evidence for a significantly improved QOL (*p*=.000) and self-efficacy (*p*=0.0075) following the use of the combined telephonic and mobile app intervention offered to CRC patients for 6 months following primary cancer surgery. Another study found that HRQoL and sense of coherence levels remained stable throughout the chemotherapy cycles, with no statistically significant change [33].

Psychological morbidities, such as anxiety, depression, fear of cancer recurrence (FCR), distress and fatigue, were reported in eight studies. Both Avci et al. [26] and Beaver et al. [28] used the State-Trait Anxiety Inventory to measure CRC patients’ anxiety levels during eHealth follow-up. They found that receiving a web- and telephone-based eHealth educational intervention, counselling and support [26] and a nurse-led telephone-based intervention comprising CRC care information and inquiring symptoms and emotional concern had significant effects [28]. Anxiety and depression were measured by the Hospital Anxiety and Depression Scale (HADS) in the context of a telephone-based intervention of education and support, and both anxiety and depression levels decreased significantly, with *p*=.07 and .037, respectively, compared with controls [38]. A similar telephone-based intervention significantly reduced depression levels (*p*≤.05) in CRC patients who received reminiscence therapy in addition to telephone-based support, but this result was not achieved with standard care and telephone support only [30]. FCR was included as a PROM in one pre-post-longitudinal intervention using telemedicine applications and showed no statistically significant differences from baseline to the 12-week timepoint [34]. In one telephone-support-based study [51] and one using a range of remote follow-up approaches (e.g. smartphone app, messaging) [29], no significant time or group differences in distress were observed. Only one study included fatigue as a PROM, measured by the Functional Assessment of Cancer Therapy-Fatigue (FACT-F) scale. Fatigue changed in the hypothesised direction but did not reach statistical significance [36].

Three studies reported on how eHealth may ease cancer treatment side effects. The two quantitative studies by Drott et al. [32, 33] measured neurotoxicity from chemotherapy with oxaliplatin. The authors found that none of the participants returned to baseline function after the self-reporting of symptoms on a mobile phone-based system. Avci et al. [26] found that the intervention group experienced chemotherapy side effects significantly less frequently compared with the control group (*p*≤.05), as well as less severe side effects of infection, hair loss and mouth and throat problems (*p*≤.05), after receiving a web- and telephone-based eHealth intervention of education, counselling and support.

In six studies, health behaviours such as PA, sedentary behaviour and a healthy diet were studied as outcomes of eHealth interventions. Self-reported PA was included in five of them. All five found significant improvements in PA after CRC patients received eHealth interventions in the form of smartphone applications with information, real-time communication and monitoring [29, 35, 42] or telephone consultations and written information [36, 43]. Sedentary behaviour was explored in the RCT by Lynch et al. [40]. They found that telephone-based counselling, including exercise instructions and regular support from intervention staff, did significantly reduce sedentary behaviour in a sample of CRC patients. However, no significant differences were observed between the intervention and control groups [40]. Two studies investigated the benefits of eHealth interventions regarding patient diet or nutritional status using a smartphone application [29] or telephone consultations and written information [36]. Only Grimmett et al. [36] succeeded in proving that the eHealth intervention was beneficial, showing significant increases in self-reported fruit and vegetable consumption (*p*<.001), with the mean intake exceeding seven portions a day at follow-up. In addition, red meat intake was significantly reduced (*p*<.013).

Self-management was reported in three of the included articles, representing two studies [47–49]. Here, the authors used the patient activation measure to study CRC patients’ self-management knowledge, skills and confidence. They found no statistical difference between the intervention group following the Oncokompas intervention for 6 months and the control group [47–49]. Four studies described the eHealth intervention as promoting the self-management of CRC patients but did not include self-management as an outcome [33, 36, 37, 49]. In Qaderi et al., self-management information was included as part of a cost overview and as a measurement of CRC patients’ satisfaction with the self-management content of a remote follow-up service following surgery [44].

### Patient experiences of eHealth follow-up interventions

A total of five studies explored CRC patients’ experiences with using eHealth as part of follow-up during or after cancer treatment [27, 32, 44, 46, 50]. Van Blarigan et al. [46] assessed the acceptability of a PA intervention by surveying the participants’ access rates to a Fitbit website and use of interactive text messages. In this study, the intervention was perceived as highly acceptable, and the text messages were found to motivate the participants to exercise. In two other studies, video consultations were used as part of remote follow-up care for CRC patients. Barsom et al. [27] found that video consultations were highly valued for being easy and convenient to use and that the majority of participants wanted to use video consultations in the future. In Qaderi et al. [44], CRC patients expressed high levels of satisfaction with receiving remote follow-up that resulted in fewer hospital visits, saved time and costs, increased healthcare accessibility and efficiency and better communication with healthcare professionals. The perceived disadvantages included less frequent face-to-face contact with healthcare professionals.

The only aim of both qualitative studies was to explore CRC patients’ experiences with eHealth. In Drott et al. [32], CRC patients’ experiences of using a mobile phone-based system to report symptoms were identified and constructed as the patients being involved in their own care by observing treatment side effects, being able to choose the time and place they answer the questions, and gaining knowledge on how side effects can vary during the cycles of treatment. All the patients in Williamson et al. [50] found telephone follow-up (TFU) to be a positive experience, and all stated a preference for continuing with TFU. They experienced TFU as being accessible, convenient and personalised, and their relationship with the specialist nurse was well taken care of through the telephone consultations. A summary of findings on patient experience with eHealth interventions is displayed in Table 3.

## Discussion

This integrative review aimed to review the research on eHealth interventions in the context of CRC survivorship published over the past 10 years to evaluate the mode of delivery, user interface and content of various eHealth interventions, patient adherence to the intervention, effects of eHealth interventions on PROMs and patients’ experiences of eHealth follow-up interventions.

Post-surgery eHealth interventions for the follow-up of CRC patients largely revolved around a telephone-based user interface, mainly delivering information, advice or support. For patients with low digital competence or without access to technology, simple solutions like the telephone appear preferable. Meanwhile, it is important to continue to develop more advanced technology that requires patients to play an active role in its application. A substantial number of smartphone subscriptions worldwide enable the use of mHealth applications to support and guide patients with cancer towards better self-management and improved health literacy [52].

The monitoring of health, symptoms or health behaviours was included in all the studies, while over 50% of them provided remote education and information through eHealth. CRC patients report extensive information needs post-surgery and need self-management support to avoid complications and restore normalcy [10]. To support CRC patients’ self-management needs, a blended approach to eHealth is suggested, with more involvement and attention from healthcare professionals in combination with the technology to ensure successful implementation [53].

Eleven of the studies addressed patients’ adherence to the eHealth interventions, and the range among those that calculated adherence rates was 62 to 100% [29, 32, 35, 36, 40–42, 44, 46, 49, 51]. This is in line with earlier research on cancer patients’ encounters with digital health interventions, which showed adherence rates between 70 and 100% [14]. The included studies that reported on patients’ adherence to eHealth interventions mainly focused on patient uptake of the intervention, and none of them provided a definition based on a theoretical understanding of adherence. The successful implementation of eHealth services relies on systematic evaluations founded on theoretical frameworks [54]. Moreover, uptake of eHealth interventions is aided by the fact that approximately 85% of the global population are connected through mobile networks, with a 5-year increase in smartphone users of 5% [55].

A clear link between eHealth adherence and technology acceptance is predicted by perceived usefulness and perceived ease of use [56]. In this review, patients were found to perceive eHealth interventions as highly acceptable and valuable, with both intervention-related factors and patients’ personal gains applying to the CRC patients’ opinions. Among the intervention-related factors that may positively influence cancer patients’ adherence to eHealth are content tailored to meet the patient’s needs, customised reminders and real-time contact with healthcare professionals [57].

A cancer diagnosis threatens a patient’s emotional health. We found evidence for improved mental health outcomes resulting from eHealth follow-up, mainly decreased anxiety and depression levels [28, 30]. As confirmed in earlier reviews, eHealth approaches can manage psychological distress among cancer patients [58]. An essential finding of this review is the lack of follow-up on FCR among CRC patients, studied in only two of the eHealth interventions. Healthcare professionals need to recognise and support CRC patients’ FCR in the early post-treatment stages. eHealth interventions offered during the transition from hospital to home may provide strategies to manage fear and improve the patient’s help-seeking behaviour [59].

Many cancer survivors experience physical symptoms that limit their daily life activities and decrease their QOL, and the integration of survivorship-centred care is crucial throughout the cancer care trajectory [60]. In the current review, evidence of the efficiency of eHealth in ameliorating chemotherapy side effects was weak. Interestingly, benefits of eHealth follow-up were observed when web-based counselling and education were paired with TFU, involving human contact [26]. Considering the finding that CRC patients highly appreciate the combination of personalised care via eHealth programs and sufficient communication with healthcare professionals through eHealth, we argue that eHealth solutions without any human interaction may prove less valuable. This statement is supported by an overview of 15 reviews during post-treatment cancer survivorship care, recommending hybrid approaches combining telemedicine with face-to-face support [16].

Cancer patients may experience a ‘teachable moment’ in the wake of a cancer diagnosis, leading to a change in health behaviours [61] that depends on motivational support from healthcare professionals [62]. We found that eHealth interventions containing information, monitoring and real-time communication from healthcare professionals improved CRC patients’ engagement in PA. The monitoring of behaviour is the cornerstone of a health behaviour change and is often associated with a positive result [63]. In addition, eHealth may facilitate participation for cancer patients who lack access to or cannot conveniently access PA programs in their community [64].

## Strengths and limitations

This review clearly describes the methods and outlines the process of data identification and selection as well as steps to synthesise the results from individual studies and evaluate the evidence, all of which create a robust and meaningful review. The inclusion of studies with different study designs enabled a more comprehensive approach to meeting the study aims. On the other hand, even though we employed a rigorous literature search overseen by a highly experienced librarian and used a digital sorting tool for the screening of records, relevant records may have been missed. We did not exclude inadequately reported studies as doing so would not affect the findings in any meaningful way [64].

## Conclusion

In this review, we identified 26 studies of eHealth interventions following the discharge of patients from the hospital after curative surgery for CRC. eHealth interventions upon hospital discharge can offer support during a critical period. This review demonstrated that eHealth interventions were mainly telephone-based, delivering education, counselling or support and monitoring symptoms or health behaviours. However, there was a lack of focus on CRC patients’ adherence to eHealth. More research is needed on adherence to eHealth programs and its relationship with the implementation of eHealth in CRC populations.

eHealth follow-up may mitigate anxiety and depression in CRC patients, while the proof of its impact on other psychological morbidities or QOL is less clear. We also did not find strong evidence of the ameliorating effects of eHealth programs regarding the side effects of cancer treatment. eHealth interventions may have a positive influence on CRC patients’ PA behaviours regardless of the user interface, but the combination of technology and human interaction appears important. In general, remote, digital follow-up was experienced as positive, accessible and usable and as an improvement to healthcare services delivery.

This review can inform future intervention research on discharge planning in CRC care. In addition, it may support clinicians working towards ensuring the uneventful and swift recovery of CRC patients. Furthermore, the findings may have value in the development of eHealth services for other cancer patient populations.

## Declarations

### Disclaimer

The views expressed are those of the author and not necessarily the NHS, NIHR, or Department of Health and Social Care.
